# Three operational taxonomic units of *Eimeria* are common in Nigerian chickens and may undermine effective molecular diagnosis of coccidiosis

**DOI:** 10.1186/s12917-016-0713-9

**Published:** 2016-06-04

**Authors:** Isa D. Jatau, Idris A. Lawal, Jacob K. P. Kwaga, Fiona M. Tomley, Damer P. Blake, Andrew J. Nok

**Affiliations:** Faculty of Veterinary Medicine, Ahmadu Bello University, Zaria, Nigeria; Pathology and Pathogen Biology, Royal Veterinary College, Hawkshead Lane, North Mymms, AL9 7TA UK

**Keywords:** *Eimeria*, Chickens, Nigeria, Poultry, Prevalence, Operational taxonomic unit

## Abstract

**Background:**

Chicken is fast becoming the world’s most consumed meat. As a consequence poultry health is more important now than ever before, with pathogens of chickens recognised as serious threats to food security. One such threat are *Eimeria* species parasites, protozoa which can cause the disease coccidiosis. *Eimeria* can compromise economic poultry production and chicken welfare, and have serious consequences for poor livestock keepers. Seven *Eimeria* species that infect chickens are recognised with a global enzootic distribution. More recently three cryptic Operational Taxonomic Units (OTUx, y and z) have been described in populations of *Eimeria* recovered from chickens in Australia. Two of the three OTUs have also been detected in sub-Saharan Africa, but their occurrence, pathology and the risk they pose is largely unknown.

**Results:**

Nigeria has witnessed a dramatic expansion in poultry production and is now the largest poultry producer in Africa. Here, faecal samples collected from nine of 12 commercial chicken farms sampled in Kaduna state, Nigeria, were found to contain eimerian oocysts. After amplification by in vivo propagation all three cryptic OTU genotypes were detected using polymerase chain reaction (PCR), including OTUy for the first time outside of Australia. Comparison with a widely used, established *Eimeria* species-specific PCR assay revealed failure to detect the OTU genotypes.

**Conclusions:**

All three of the *Eimeria* OTU genotypes appear to be common in north-western Nigeria. The failure of a leading species-specific molecular assay to detect these genotypes indicates a risk of false negative *Eimeria* diagnosis when using molecular tools and suggests that the spatial occurrence of each OTU may be far wider than has been recognised. The risk posed by these novel genotypes is unknown, but it is clear that a better understanding of *Eimeria* occurrence is required together with the validation of effective diagnostics.

**Electronic supplementary material:**

The online version of this article (doi:10.1186/s12917-016-0713-9) contains supplementary material, which is available to authorized users.

## Background

Poultry farming is one of the fastest growing sectors of animal production in the world with the greatest increases occurring in developing countries in Africa and Asia [1]. The expansion is predicted to continue for at least 30 years, posing enormous challenges for system development and pathogen control [2]. In Africa, including Nigeria, many people depend on small-scale poultry production systems for food, currency, work and business, but diseases such as coccidiosis are a major limiting factor [3, 4]. Coccidiosis, caused by protozoan *Eimeria* species parasites, is a major recurring disease which exerts a negative effect on profitable and sustainable small-scale poultry enterprises around the world, including in Africa [5]. Seven recognized species of *Eimeria* specifically parasitize the digestive tract of domestic chickens (*Gallus gallus domesticus*) [6]. *Eimeria acervulina, Eimeria maxima*, *Eimeria necatrix* and *Eimeria tenella* are commonly considered to be most important as a consequence of their occurrence, fecundity and pathogenicity, but all species can undermine chicken health and welfare [2, 6, 7]. Concurrent infection with multiple *Eimeria* species is common and clinical manifestation of the disease can vary substantially [8]. Reports of three genetic variants (termed *Eimeria* operational taxonomic units or OTUs x, y andz) circulating among poultry in Australia has added further complexity [9, 10], with two of these variants also detected recently in sub-Saharan Africa [5]. Morphological descriptions of OTU genotype oocysts are not currently available. Preliminary indications from limited genetic resources suggest OTUx and OTUy are most likely to be divergent strains of *E. maxima* and *Eimeria brunetti* respectively, while OTUz remains annotated as a cryptic species with its genetic relatedness to the seven recognised species unclear [5, 9] and [Clark et al., manuscript submitted].

Reports on the occurrence of *Eimeria* species in Nigerian chickens have previously been based on traditional protocols using morphological and pathological criteria in the diagnosis of coccidiosis [11, 12]. While effective, these traditional methods can be subjective and unreliable in the diagnosis of natural infections, especially during sub-clinical infection when more than one species is present [13]. Consequentially, the use of molecular tools for accurate species identification and characterization of regional isolates of this parasite is valuable. Using molecular tools can reduce or obviate the requirement for time-consuming, and frequently subjective microscopic analysis, but relies on sequence conservation within the genomic regions targeted by the assays. The impact of genetic variation between isolates of the same species on PCR detection has been described previously for both *E. maxima* and *Eimeria mitis*, where multiple primer sets have been required for some targets [14]. The ability of many current molecular tools to identify parasites of the OTU genotypes is untested and may represent a risk of under-detection. PCR assays developed by Fornace and colleagues [5], and Godwin and Morgan [15] have attempted to address this deficit, although the latter requires equipment that is not currently widely available in many laboratories. Effective detection of circulating *Eimeria* species is important for proper diagnosis and disease control, and can inform the selection of appropriate anticoccidial drugs and vaccines [16, 17]. All seven of the recognised *Eimeria* species have been detected previously in Nigeria [12]. Here, we report the use of species-specific molecular assays to document the occurrence of all three *Eimeria* OTU genotypes in Nigeria with relevance to poultry husbandry and health.

## Results

### Morphological identification of *Eimeria* species oocysts in commercial poultry

Twelve farms found during previous routine veterinary monitoring to harbour coccidial oocysts were sampled. Oocysts were assigned putative species identity based upon microscopic morphology [18]. Briefly, small oocysts were categorised as *E. acervulina* and/or *E. mitis* (group AM, oocysts ≤18.8 μm long), medium sized oocysts as *E. necatrix*, *E. tenella* and/or *Eimeria praecox* (group NTP, 18.9-23.8 μm long) and larger oocysts as *E. brunetti* and/or *E. maxima* (group BM, ≥23.9 μm long). Nine samples were found to contain oocysts which were capable of sporulation, all of which contained medium sized oocysts consistent with the occurrence of NTP *Eimeria* species (75 % of the samples tested). Large oocysts, indicative of *E. brunetti* and/or *E. maxima*, were also observed in all nine oocyst positive samples (75 %). Small oocysts, representing *E. acervulina* and/or *E. mitis*, were detected in eight samples (70 %; Table 1).

**Table 1:**
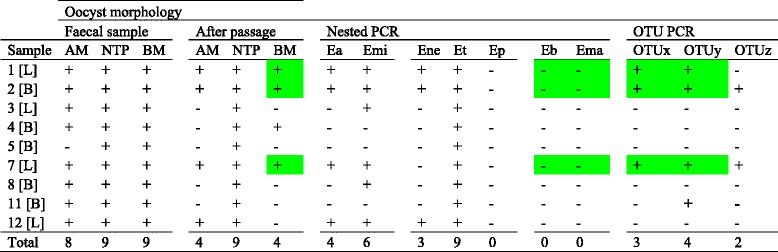
Identification of *Eimeria* species and OTU genotypes recovered from commercial poultry farms in Zaria

*Eimeria* occurrence was determined by oocyst morphology before and after in vivo propagation, and species/genotype-specific PCR after propagation. Highlighted boxes indicate samples found to contain large oocysts by microscopy after passage and OTUx and y genotypes, but not *E. brunetti* or *E. maxima* by PCR. Ea = *E. acervulina*, Eb = *E. brunetti*, Ema = *E. maxima*, Emi = *E. mitis*, Ene = *E. necatrix*, Ep = *E. praecox*, Et – *E. tenella*. Morphological identification: AM = small oocysts (Ea, Em), NTP = medium oocysts (En, Et, Ep), BM = large oocysts (Eb, Ema). L = layer breed chicken (ISA Brown), B = broiler breed chicken (Cobb). + = parasite detected, - = parasite not detected

Oocysts recovered from all nine positive samples were amplified by in vivo propagation because the original samples yielded low oocyst recovery and poor sporulation. Oocysts were harvested at post-mortem directly from the caeca to enrich the putative *E. tenella* sub-population of each sample for related studies [19]. As a consequence, the species complexity was reduced in five of the nine samples (Table 1). Specifically, only four of eight previously positive samples retained detectable AM size oocysts, and four of nine samples retained BM sized oocysts. NTP sized oocysts persisted in all nine samples.

### Occurrence and identification of *Eimeria* using nested species-specific PCR

Nested PCR targeting the seven recognised *Eimeria* species confirmed the occurrence of *E. tenella* in all NTP oocyst positive samples (Table 1). *Eimeria praecox* was not detected, but three of the nine samples were also found to contain *E. necatrix*. All four samples found to contain AM morphology-group oocysts were shown to include both *E. acervulina* and *E. mitis* genomic DNA, with two AM negative groups also found to contain *E. mitis* by nested PCR. In direct contrast, all four samples found to contain BM morphology-group oocysts were negative for *E. brunetti* and *E. maxima* genomic DNA by nested PCR (Table 1). Two amplicons representative of each species detected were cloned and sequenced (accession numbers LT549029-LT549036). BLASTn comparison against the GenBank non-redundant database confirmed species identity for each assay.

### Detection of *Eimeria* OTU genotypes by ITS-2 PCR

All three OTU genotypes were detected by OTU-specific PCR targeting the internal transcribed spacer (ITS) 2 sequence from mixed populations of oocysts collected in Nigeria following in vivo passage and caecal harvest [5]. OTUy was most common, detected in four of nine samples screened (44.4 %). The OTUx genotype was detected in three of nine samples (33.3 %), and always in the presence of the OTUy genotype. All three OTUx and OTUy positive samples had previously been found to contain large (BM morphology-group) oocysts (Table 1). The OTUz genotype was detected in two of nine samples screened (22.2 %), both of which were also found to contain OTUx and OTUy genotypes. Two amplicons representative of each OTU genoptype were cloned and sequenced (accession numbers LT549037-LT549042). BLASTn comparison against the GenBank non-redundant database confirmed genotype identity for each assay.

## Discussion

The original objective of these studies was to recover *E. tenella* isolates from Nigerian poultry as a step towards population genetic characterisation [19]. For this reason samples were initially screened microscopically for the occurrence of oocysts of the NTP size and type (i.e. medium sized oocysts) [18]. The regional prevalence of each *Eimeria* species and genotype has not been calculated since the sample size was limited and the choice of farms sampled was biased, focusing on farms previously identified as coccidia positive. Nonetheless, the range of occurrence of each species was comparable to that described previously in the study area and in the southern part of Nigeria [12, 20, 21], possibly influenced by collection during the wet season when litter oocyst levels were likely to be elevated [22]. The reduction in *Eimeria* species complexity detected in many of the samples after in vivo propagation is likely to be a consequence of harvest by caecal, rather than faecal oocyst recovery 7 days post infection. The detection of *Eimeria* species not known to replicate in the caeca is likely to represent oocysts produced higher up the gastrointestinal track and transiting through the caeca at the time of sampling.

*Eimeria* of the OTUx and OTUz genotypes have previously been detected in sub-Saharan Africa, but until now the OTUy genotype has only been described in Australia [5, 9, 10]. Here, analysis of a small number of field isolates clearly indicated for the first time that all three OTU genotypes are circulating within Nigerian poultry. Four of nine samples tested were found to contain at least one OTU genotype (44 %), which is comparable to the occurrence detected within Australian flocks [10]. The risk posed by these genotypes is currently unclear. Limited evidence indicates that live vaccines such as Eimeriavax® 4 M, formulated to include *E. acervulina*, *E. maxima*, *E. necatrix* and *E. tenella*, cannot prevent colonisation by OTU genotype parasites under field conditions [23]. It is likely that current anticoccidial prophylaxis will be capable of controlling these parasites, but it is equally likely that they will be capable of developing anticoccidial resistance as has been shown for other *Eimeria*/drug combinations [24]. The absence of severe disease associated specifically with OTU genotype parasites in Australia indicates that current control measures remain adequate, but as the range of anticoccidial drugs available is reduced, live and possible future subunit or recombinant vaccines may be compromised.

Comparison of microscopic observation of oocysts with PCR-based detection of *Eimeria* genomic DNA yielded variable results. NTP size-type oocysts were detected in all nine samples by microscopy and were confirmed by the detection of *E. necatrix* and/or *E. tenella* genomic DNA. *Eimeria praecox* genomic DNA was not detected and these parasites may not have been present, although this species reproduces in the duodenal loop with a prepatent period of just 84 h and may have been lost if present during caecal harvest occurring 7 days post infection [25]. AM size oocysts were detected in four of the nine samples tested following propagation through chickens. Molecular analysis confirmed the presence of *E. acervulina* and/or *E. mitis* genomic DNA in these samples and detected two further positive samples, possibly indicating greater sensitivity, although it should be noted that the PCR employed would have detected parasite genomic DNA from all lifecycle stages and would not have been restricted to the oocyst. The greatest inconsistency was detected for BM size-type oocysts. Oocyst detection indicated the presence of BM oocysts in four of the nine samples tested, but all were PCR negative for *E. brunetti* and *E. maxima*. Such variation between the microscopic and molecular approaches may have been associated with the limit of detection for the PCR assay, but the finding that three of the four BM oocyst positive samples contained genomic DNA of the OTUx and OTUy genotypes suggests that current molecular diagnostics are inadequate in regions where these parasites may be circulating. Thus, the true extent of the occurrence of OTU genotype *Eimeria* is unknown.

## Conclusions

Application of molecular diagnostics for the occurrence of the seven recognised *Eimeria* species and OTU genotypes has detected all three OTUs circulating in Nigerian poultry. Inconsistencies between microscopic and molecular diagnostic approaches indicate that molecular tools require updating to include the OTUx, y and z genotypes. This is the first report of the OTUy genotype outside of Australia.

## Methods

### Study area

Kaduna state is among the seven states of the north-western geopolitical zones of Nigeria. It is situated within the Sudan savannah vegetation zones of Nigeria with distinct dry and wet seasons. The dry season runs from October to April. The wet season begins in most parts of the state in May and lasts up to September or October, with mean annual rainfall of between 510–1140 mm. The samples analysed here were collected from poultry units around Zaria, Nigeria, between April and July 2013.

### Farm selection and sample collection

Combined faecal/litter samples representative of a pen, and thus several individual chickens, were collected from 12 commercial poultry farms found during previous routine veterinary monitoring to harbour coccidial oocysts. In each poultry house, samples were collected following an approximate W-shaped path, starting and finishing in the corners of one of the long sides of the house [3]. Along this path, combined faecal/litter samples were collected manually, stopping every three strides to collect one handful which was placed in clean plastic bags and transported on ice pack to the Research Laboratory of the Department of Veterinary Parasitology and Entomology, Ahmadu Bello University-Zaria.

### Sample processing and morphometric oocyst identification

In the laboratory, 200 g of each sample was weighed, transferred into a plastic beaker and soaked in approximately 500 ml of distilled water overnight. The soaked samples were then homogenized by thorough stirring using a glass rod and filtered through a metal sieve (mesh size 300-500 μm). The filtrate from each sample was allowed to sediment for one hour on the laboratory bench, after which the supernatant fluid was discarded into a clean beaker. The presence of oocysts was confirmed microscopically by transferring the equivalent of 10 ml of sediment into a centrifuge tube and testing for the presence of coccidial oocysts using the saturated saline flotation technique described elsewhere [26]. Oocysts were assigned putative species identity based upon microscopic morphology [18]. For each positive sample, oocysts were recovered from the remaining sediment using the centrifugal flotation technique [26]. The harvested oocysts were re-suspended in distilled water and washed by centrifugation three to four times to remove the flotation solution (300 g for 5 min). The sediment containing the oocysts was transferred into Petri-dishes, re-suspended in 2.5 % (w/v) potassium dichromate solution and allowed to sporulate at room temperature for 7 days with regular stirring.

After sporulation, oocysts within each sample were cleaned from the residual faecal debris by treatment with sodium hypochlorite (4 % active chlorine) and three successive washes in distilled water as described elsewhere [27]. After cleaning, the oocysts were re-suspended in distilled water and enumerated using a modified-Fuchs Rosenthal counting chamber. The tubes containing the cleaned oocysts were clearly labelled with isolate number, date and amount of oocysts/ml, and stored at 4 °C until required.

### *Eimeria* propagation

Field samples found to contain coccidial oocysts which had sporulated were used for in vivo propagation as a consequence of overall low oocyst recovery and poor sporulation. Individually caged 4 week old specific-pathogen free (SPF) Light Sussex chickens were inoculated orally with 4,000 sporulated mixed oocysts from single field parasite populations. Progeny oocysts were recovered from caecal tissue and contents collected during post-mortem 7 days post infection, sporulated and purified as described elsewhere [25, 27].

### Total genomic DNA extraction

Four millilitres of each washed oocyst suspension, containing between 2 and 5 million oocysts after in vivo propagation, were centrifuged (750 g for 10 min) to pellet the oocysts. Each pellet was re-suspended in the minimum volume residual supernatant and transferred to a 1.5 ml screw top plastic tube. Glass beads (0.4-0.6 mm; Sigma, UK) equivalent to the volume of the oocyst pellet were added to the tube and covered with sterile phosphate buffered saline (PBS; pH 8.0). The pelleted oocysts were then disrupted using a Mini Beadbeater-8, (Biospec Products, Bartlesville, USA) for two minutes and total genomic DNA (gDNA) was isolated from the smashed oocyst homogenate using a QIAamp DNA Tissue mini kit (Qiagen, Germany) following the manufacturers protocol.

### Molecular identification of *Eimeria* by nested polymerase chain reaction

A standardized nested PCR assay targeting the internal transcribed spacer (ITS)-1 sequence for identification of *Eimeria* species of poultry was used to improve detection of minority *Eimeria* species populations. Primers amplifying the entire ITS-1 sequence based in the flanking 18S and 5.8S rDNA regions of the eimerian genome were used in the first genus-specific PCR phase, while species-specific primers targeting the ITS-1 region were used to amplify the individual *Eimeria* species in the second nested phase. The primers (as shown in Additional file 1) and the PCR conditions used were as described previously [3, 14]. Genomic DNA purified from the Houghton reference strains of each of the seven recognised *Eimeria* species were used as positive controls, with molecular grade water (Sigma, UK) used as the negative control starting from the beginning of the nested assay. The amplification products of the specific nested PCR were analysed by gel electrophoresis in 2 % (w/v) agarose gels in 1x Tris Acetate EDTA (TAE; all Sigma, UK) buffer stained with 0.01 % (v/v) SafeView nucleic acid dye (NBS Biologicals, UK).

### Molecular identification of new operational taxonomic unit (OTU) cryptic *Eimeria* genotypes

Primers specific to the OTUx, OTUy and OTUz ITS-2 sequences were used to screen each sample for the occurrence of these novel genotypes (Additional file 1). PCR conditions were as described previously and the PCR products were analysed by gel electrophoresis as described above [5].

### PCR amplicon sequencing to confirm and validate species/genotype identification

Two PCR fragments representative of each *Eimeria* species detected were sequenced to confirm amplicon identity and validate PCR detection, resulting in 14 sequences from 31 positive reactions (45 %). Amplicons were purified using a Qiagen PCR purification kit, cloned using pGEM-T Easy (Promega, Madison, USA) in XL1-Blue MRF *Escherichia coli* (Stratagene, La Jolla, USA), miniprepped (Qiagen) and sequenced (GATC Biotech, Konstanz, Germany) as described by the respective manufacturers. Sequence assembly, annotation and interrogation were undertaken using CLC Main Workbench v6.0.2 (CLC Bio, Katrinebjerg, Denmark) and sequences were identified using BLASTn against the GenBank non-redundant database with default parameters. The sequences have been submitted to GenBank under the accession numbers LT549029-LT549042.

## Additional file

Additional file 1: Polymerase chain reaction (PCR) primers used for molecular identification of *Eimeria* species and OTU genotypes. (DOCX 23 kb)
