# Supplementary material for: Three operational taxonomic units of Eimeria are common in Nigerian chickens and may undermine effective molecular diagnosis of coccidiosis
Source: BMC Vet Res. 2016 Jun 4;12:86. doi: 10.1186/s12917-016-0713-9 (PMC4893269; doi:10.1186/s12917-016-0713-9)
Supplement: Additional file 1: — Polymerase chain reaction (PCR) primers used for molecular identification of Eimeria species and OTU genotypes. (DOCX 23 kb) [file 12917_2016_713_MOESM1_ESM.docx]

**Additional file 1:** Polymerase chain reaction (PCR) primers used for molecular identification of *Eimeria* species and OTU genotypes.

| Assay | Primer | Target | Sequence (5' - 3') |
| --- | --- | --- | --- |
| Nested ITS-1 | EF1 | *Eimeria* ITS-1 | aagttgcgtaaatagagccctc |
| (Lew *et al.*, | ER1 |  | agacatccattgctgaaag |
| 2003) | EAF | *Eac* ITS-1 | ggcttggatgatgtttgctg |
|  | EAR |  | cgaacgcaataacacacgct |
|  | EBF | *Ebr* ITS-1 | gatcagtttgagcaaaccttcg |
|  | EBR |  | tggtcttccgtacgtcggat |
|  | EMRA1 | *Ema* US ITS-1 | gtgat/atcgttc/tgg/ag/aagtttgc |
|  | EMFA1 |  | ct/acaccactcacaatgaggcac |
|  | EMRA2 | *Ema* Aus ITS-1 | gcggtttcatcatccatcatcg |
|  | EMRA2 |  | cgttgtgagaag/aactga/gaaggg |
|  | EMi1FA | *Emi* 1 ITS-1 | gggtttatttcctgtcc/gtcgtctc |
|  | EMi1RA |  | gcaagagagaatcggaatgcc |
|  | EMi5FA | *Emi* 5 ITS-1 | cggagctggggttttctttc |
|  | EMi5Ra |  | cctgcatatccaca/gtt/cgaac/atac |
|  | ENF | *Ene* ITS-1 | tacatcccaatctttgaatcg |
|  | ENR |  | ggcatactagcttcgagcaac |
|  | EPRA | *Epr* ITS-1 | ccaagcgatttcatcatt/cgggga/g |
|  | EPFA |  | aaaa/gcaa/cagcgattcaag |
|  | ETF | *Ete* ITS-1 | aatttagtccatcgcaaccct |
|  | ETR |  | cgagcgctctgcatacgaca |
|  |  |  |  |
| *Eimeria* OTU | OTU-Xfor | OTUx ITS-2 | GTGGTGTCGTCTGCGCGT |
| (Fornace *et al.*, | OTU-Xrev |  | ACCACCGTATCTCTTTCGTGA |
| 2013) | OTU-Yfor | OTUy ITS-2 | CAAGAAGTACACTACCACAGCATG |
|  | OTU-Yrev |  | ACTGATTTCAGGTCTAAAACGAAT |
|  | OTU-Zfor | OTUz ITS-2 | TATAGTTTCTTTTGCGCGTTGC |
|  | OTU-Zrev |  | CATATCTCTTTCATGAACGAAAGG |
